# Challenges to ethical public engagement in research funding: a perspective from practice

**DOI:** 10.12688/openreseurope.18126.2

**Published:** 2024-11-06

**Authors:** Kalli Giannelos, Martijn Wiarda, Neelke Doorn

**Affiliations:** 1 Governance and Regulation Chair, University of Paris Dauphine-PSL, Paris, Île-de-France, 75016, France; 2 Centre for Political Research, Sciences Po, Paris, Île-de-France, 75007, France; 3 Faculty of Technology, Policy and Management, Delft University of Technology, Delft, The Netherlands

**Keywords:** engagement, participation, responsible research and innovation, research funding, ethics, public engagement

## Abstract

European research funding organizations (RFOs) are increasingly experimenting with public engagement in their funding activities. This case study draws attention to the challenges they face in preparing, implementing, and evaluating ethical public engagement in the context of setting funding priorities, formulating calls for proposals, and evaluating project proposals. We discuss challenges related to seven themes: (1) recruiting participants; (2) commitments and expectations; (3) meaningful dialogue and equal engagement; (4) accommodating vulnerability; (5) funding call formulations; (6) lack of expertise in engagement ethics; and (7) uncertainty, resource constraints, and external factors. To address these challenges, we propose the following seven interventions: (1) developing comprehensive recruitment strategies with experienced recruiters and community organizations; (2) establishing clear communication of roles, expectations, and outcomes through codes of conduct; (3) training mediators to address power imbalances; (4) designing flexible engagement methods and providing tailored support; (5) implementing collaborative feedback loops for inclusive funding call formulation; (6) enhancing ethical standards through internal expertise and external advisory inputs; and (7) developing adaptive strategies for flexible and ethical public engagement. These recommendations emphasize the need for context-adaptive insights to support funding organizations to implement ethical public engagement activities, even when faced with organizational constraints and a lack of ethical expertise.

## Introduction

Research funding organizations (RFOs) across Europe are increasingly experimenting with forms of public engagement (e.g. citizen panels) in their efforts to set funding priorities, formulate funding calls, and evaluate project proposals ( Den Oudendammer *et al.*, 2019; Rowe *et al.*, 2010). In this paper, ‘public engagement’ refers to the interaction with non-traditional stakeholders, such as citizens and civil society organizations ( Dreyer *et al.*, 2021; Kahane *et al.*, 2013), in research and innovation activities led by RFOs. We recognize that the term may vary in meaning across different sectors, disciplines, and countries, but for clarity, we use ‘public engagement’ to encompass all forms of involvement RFOs might undertake. These experiments focus on including ‘non-traditional’ stakeholders such as citizens, communities, and civil society organizations, alongside ‘traditional’ ones like researchers, experts, and policymakers. These engagement processes mainly take the forms of communication, consultation, and participation, granting the public a certain degree of power ( Arnstein, 1969; Rowe & Frewer, 2000; Rowe & Frewer, 2005). However, communication and consultation are typically top-down processes, where public empowerment remains limited and participation is often more symbolic than substantive ( IAP2, 2018).

Despite the potential of public engagement, several challenges hinder its success: these include issues related to recruitment, engagement ethics, and managing public expectations. Additionally, variations in research governance models across regions affect how and by whom engagement activities are conducted ^1^. However, when executed effectively, public engagement can lead to more competitive and desirable outcomes ( Fraaije & Flipse, 2020) and allow RFOs to explore societal values and worldviews that cannot be determined top-down or *a priori* ( Bauer *et al.*, 2021). Although the public may lack technoscientific expertise, they often possess complementary know-how and context-specific experiences that are crucial for solving societal challenges ( Ravetz, 1999). For instance, informal caregivers may offer valuable insights into innovations that could improve a patient's quality of life: such ‘experts by experience’ can thus improve the social robustness of innovation ( Nowotny, 2003).

This emerging trend is inspired by various research fields, such as (Participatory) Technology Assessment ( Durant, 1999; Schot & Rip, 1997), Responsible Research and Innovation ( Stilgoe *et al.*, 2013; Von Schomberg, 2013; Wiarda *et al.*, 2021), Ethical Legal and Social Implications/Aspects Research ( Fisher, 2005), and more. These fields endorse upstream public engagement on the basis of various normative, instrumental, and substantive grounds ( Stirling, 2008; Wilsdon & Willis, 2004).

However, it is essential that public engagement is meaningful, fair, and effective ( Ayre *et al.*, 2018; Meskens, 2020). The uptake of engagement in research funding frequently raises ethical issues requiring explicit consideration ( Van Bekkum & Hilton, 2014). Concerns may relate to issues like exploitation, vulnerability, representation, and inequality. These concerns often do not have a clear-cut solution and are exacerbated by practical constraints. Addressing these challenges requires explicit attention in both academia and practice.

Broader insights into participatory processes across various funding activities at both local and central government levels are well-documented in the scholarly literature. These processes, such as participatory budgeting and public involvement in budget formulation, have been explored in decentralized contexts ( Nasution & Lutfi, 2022), local government practices ( O'Hagan *et al.*, 2020), and at the central government level ( Ríos & Benito, 2017). In the specific context of research funding, the majority of studies focus on public engagement in health and medical research. Scholars have examined definitions of public engagement ( van Bekkum *et al.*, 2016), how patient and public involvement can improve research design and funding applications in the health sector ( McMillan *et al.*, 2018), as well as the benefits of effectively implementing public engagement in health research funding organizations ( Richards *et al.*, 2024). Additionally, research highlights the importance of qualitative engagement to achieve rigor, representation, and reflexivity in funding initiatives ( Rolfe *et al.*, 2018), as well as challenges in involving patients in health and social care research grants ( Foster *et al.*, 2024). Studies also address patient engagement in cancer research funding allocation, including strategies for funding high-cost cancer drugs while managing trade-offs and fostering acceptance ( Bentley *et al.*, 2018; Taccone *et al.*, 2023). The disproportionate focus on health and medical research in recent studies highlights the need for exploring broader public engagement across other sectors of research and innovation funding, which this study aims to address.

To explore these public engagement challenges, eight European RFOs from Austria (FFG), Belgium (Innoviris), Czech Republic (TACR), Germany (VDI/VDE), Lithuania (RCL), Norway (RCN), Romania (UEFISCDI), and Spain (CDTI) formed a consortium called PRO-Ethics. Over four years, they exchanged experiences on the ethical preparation, implementation, and evaluation of public engagement in research funding. This paper examines the difficulties these RFOs encountered in implementing public engagement processes, by describing these challenges in depth and illustrating how they manifest in practice.

While these European RFOs identified various ‘best practices’, this case study focuses on the challenges faced during implementation. These difficulties were first identified by RFOs through self-reflections and collectively discussed during three ‘cross-learning workshops’. This paper describes these challenges in depth, how they return in practice, and suggests how they can be addressed. The aim of this paper is to encourage academic efforts that could help RFOs organize public engagement more responsibly. In the following section, we will first describe seven major challenges to public engagement, after which we illustrate some of these through the example of VDI/VDE (German RFO), before suggesting recommendations to address these challenges.

## Challenges to ethical public engagement in research funding

**Figure 1.**
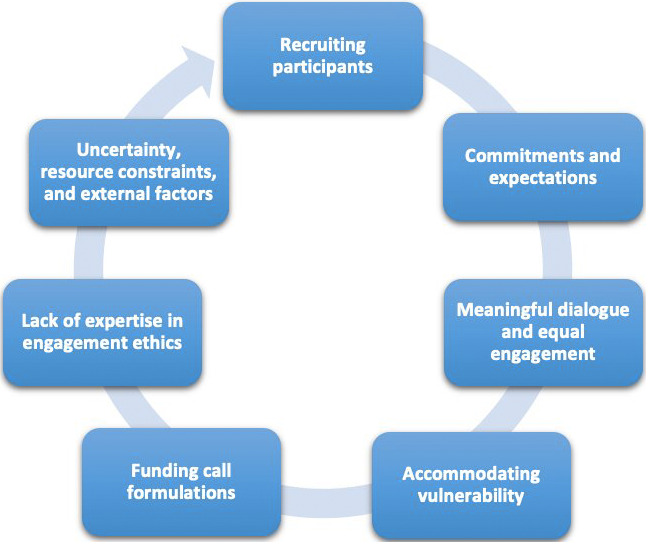
Seven main challenges identified in the ethical public engagement in research funding.

Through a series of real-life experiments, several challenges to ethical public engagement have been reported by European RFOs. These challenges relate to seven themes (see Figure 1) that will be detailed in this section. While these challenges are presented in the context of RFOs, they are not unique to this sector. Many of the issues identified are experienced across the broader research and innovation community. Researchers, institutions, and other organizations engaging the public in any stage of the research cycle face similar hurdles, making these findings relevant beyond RFOs. The research question guiding this process was to identify, at a more granular level, challenges arising from the real-life implementation of public engagement across a diverse range of research and innovation cases funded by European RFOs. The following seven challenges were identified through a series of cross-learning workshops and discussions held among the eight participating RFOs. These workshops involved self-reflections and collective analyses of public engagement processes. Data was analyzed using an inductive thematic analysis, with verbatim transcriptions coded at the sentence and paragraph levels, providing the foundation for identifying key themes and challenges.

### Challenge 1: recruiting participants

Recruiting participants for public engagement activities is a well-known challenge not only for RFOs but for the broader research community as well. RFOs strive to gather diverse public representation to ensure a wide range of perspectives in their public engagement activities ( Van Bekkum & Hilton, 2014). Defining appropriate representation for a given context is often complex, as it must take into account various socio-economic factors such as background, education, age, religion, ethnicity, and gender identity ( Den Oudendammer *et al.*, 2019).

This selection process raises several questions such as intersectionality, since individuals may identify with multiple characteristics. Potential representatives might categorize themselves based on their own understanding of their identity, leading to possible criticism and concerns about unequal treatment. The concept of ‘appropriate’ public representation is therefore highly contested, and some argue that a representation accurately reflecting society may reinforce existing societal politics ( Pratt, 2019). In some cases, an overrepresentation of minorities might even be necessary to mitigate power imbalances.

The context-specific nature of engagement means these challenges cannot be resolved with a standardized approach. Even when representation issues are addressed, potential participants may still be unwilling to engage. RFOs face a tension between which public actors should be represented and who is willing and able to participate, given their capacity and resources.

This challenge extends beyond RFOs, and there is a growing literature on how to increase diversity in public engagement across the entire research and innovation sector, covering for instance citizen science ( Brouwer & Hessels, 2019) or biomedical research ( Brown *et al.*, 2023). In health and social care, for example, recruitment often focuses on individuals with ‘lived experience’ of specific health conditions. National resources such as the UK's National Institute for Health and Care Research ( NIHR, 2021) provide practical frameworks to support inclusivity in research engagement. These strategies are relevant for any researcher or organization aiming to foster more diverse public engagement.

In practice, RFOs often use pragmatic approaches such as snowball sampling and partnering with multiplier organizations to recruit participants. While these methods can mitigate bias, they cannot fully eliminate it ( Viswanathan *et al.*, 2004). To address this, RFOs should collaborate with social scientists or independent research experts in the design of engagement processes that reduce various forms of bias and potential conflicts of interest.

### Challenge 2: commitments and expectations

Managing commitments and expectations is a significant challenge for RFOs because their goals often differ from those of the public. Participants might have different views on how much influence they should have on research funding decisions, leading to potential disagreements and causing some to decline or withdraw from engagement processes.

Even when expectations align, participants' commitments might still suffer if RFOs do not adequately accommodate their needs. Participation often depends on specific engagement forms and whether these align with participants' characteristics–such as disabilities–, and resources–such as time. For example, participants with disabilities may require particular accommodations, and those with limited time may find it hard to commit to lengthy processes.

To address these issues, RFOs find it helpful to identify and communicate everyone's needs and expectations regarding the roles, scope, purpose, process, and outcomes of the engagement before launching the process. Establishing clear codes of conduct can also help ensure that all participants understand and agree on these aspects. By explicitly addressing and aligning commitments and expectations, RFOs can foster more effective and inclusive public engagement.

### Challenge 3: meaningful dialogue and equal engagement

Ensuring meaningful dialogue and equal engagement is crucial for obtaining the public's input, but it is often challenged by heterogeneous perspectives that can lead to misinterpretation and conflict. In deliberative formats, discussions between public representatives and traditional stakeholders are particularly prone to power imbalances. Some stakeholders may dominate conversations due to their personalities, knowledge, or institutional roles, creating an imbalance (e.g., citizens vs. scientists). Such imbalances can disrupt the dialogue and limit the involvement of less dominant participants.

To address these challenges, several RFOs adopt strategies to mitigate knowledge-based power imbalances. These strategies include thematic warm-ups to prepare participants with introductory sessions on the topic to level the knowledge field and information management by selectively providing or withholding information to ensure all participants have a more equal understanding of the subject ( Zapata, 2009). Additionally, the use of neutral mediators (top-down moderation) helps guide dialogues, manage conflicts, and encourage participation from less vocal representatives ( Rubinelli & von Groote, 2017). Mediators play a key role in neutralizing power discrepancies during discussions and ensuring balanced deliberations ( Davies, 2013).

The effectiveness of these strategies hinges on the presence of mutual trust between stakeholders and the mediators. Trust ensures that dialogues are constructive and inclusive, allowing for balanced and equitable engagement.

### Challenge 4: accommodating vulnerability

Engagement challenges also extend to the inclusion of vulnerable groups ( Brown *et al.*, 2017). This issue is particularly prevalent when funding processes address real-life problems, as public actors affected by these problems may face social injustice, financial issues, or other disadvantages. In these contexts, vulnerability is difficult to define and understand ( Amann & Sleigh, 2021; Latif *et al.*, 2018). It is helpful to consider aspects contributing to participants’ vulnerability, such as their resources, capabilities, experiences, and identities.

Participants generally have a better understanding of their own vulnerabilities. Therefore, it can be beneficial for RFOs to rely on participants’ self-assessments rather than making assumptions themselves. One essential aspect of addressing vulnerability is promoting inclusion through compensation. Providing financial or non-financial compensation for participants' time and expenses is a recognized standard for public engagement. Compensation helps remove barriers to engagement, particularly for individuals from diverse socio-economic backgrounds, and ensures that vulnerable participants can contribute without facing additional financial burdens.

RFOs should adopt compensation as part of their public engagement activities, alongside other measures such as providing translators and improving accessibility. These efforts will ensure that vulnerable groups are fully included and supported throughout the engagement process.

### Challenge 5: funding call formulations

RFOs develop calls and strategies to allocate public funding to recipients such as universities, consortia, and researchers ( Lepori *et al.*, 2023). Public engagement in this context often involves formulating these funding calls and strategies. RFOs frequently struggle to meaningfully involve both traditional stakeholders (e.g., scientists) and public participants (e.g., citizens) in creating calls and strategies that are scientifically and socially relevant ( Den Oudendammer *et al.*, 2019). RFOs generally consider three options for engagement: (1) public participants suggest strategies and calls, which are then selected and scientifically embedded by traditional stakeholders; (2) traditional stakeholders propose strategies and calls, which are then selected and contextualized by public participants; or (3) the proposal, selection, contextualization, and scientific embedding are done collectively.

Each option has its own advantages and disadvantages. The experience of RFOs in our consortium indicates that collective interactions (option 3) often lead to power imbalances due to differences in social status and expertise. When public participants propose socially relevant strategies and calls, their scientific relevance is often perceived as low. Conversely, allowing traditional stakeholders to suggest strategies and calls, followed by selection and contextualization by public participants (option 2), has proven helpful. However, this approach risks turning into tokenism if public participants have limited decision-making power. Therefore, the appropriate engagement method is context-dependent.

### Challenge 6: lack of expertise in engagement ethics

Ensuring the ethical soundness of public engagement requires an expertise in both ethics and public engagement. While the associated skills and knowledge may improve engagement, RFOs often lack this expertise ( Giannelos *et al.*, 2022). However, it is important to clarify that ethical engagement does not necessarily require the creation of a specialized field of ‘engagement ethics’. Instead, ethical public engagement should be built on professionalism, fairness, transparency, and the availability of adequate resources. These principles should guide all public engagement activities, rather than relying solely on specialized ethical expertise.

While external experts, such as ethicists and facilitators, can contribute to improving engagement quality, over-reliance on this external expertise can create unnecessary barriers to participation and responsibility. All professionals involved in engagement should embrace ethical standards through values such as respect and fairness, rather than considering ethics as a specialized domain. This ensures that ethical engagement is not seen as a complex or burdensome process.

RFOs should focus on using flexible, ‘learning-by-doing’ approaches that remain open to feedback from participants, while adhering to established protocols, guidelines, and codes of conduct. Rather than creating additional layers of expertise, public engagement can be safeguarded by adopting these core principles and utilizing frameworks such as the *UK Standards for Public Involvement*. This allows ethical public engagement without imposing unnecessary burdens or specialized expertise.

### Challenge 7: uncertainty, resource constraints, and external factors

Even when the previously mentioned challenges are addressed, ethical engagement can still suffer from organizational constraints ( Amann & Sleigh, 2021). Comprehensive planning for engagement is beneficial, but RFOs often deal with high degrees of uncertainty. Nearly all RFOs in our consortium found their engagement processes more resource-consuming than initially anticipated.

Moreover, a wide range of external factors, such as regulations, significantly impact how RFOs prepare, implement, and evaluate public engagement. For example, some RFOs are required to follow strict governmental protocols that can make public engagement more rigid and less adaptable. These uncertainties, resource constraints, and external factors necessitate a high degree of organizational flexibility, which is challenging to achieve.

To navigate these issues, RFOs must develop adaptive strategies and be prepared to adjust their plans and resources as needed, ensuring that engagement processes remain ethical and effective despite external pressures.

## Challenges in practice: the case of an RFO

The seven aforementioned challenges are difficult to address by RFOs. In relation to the first challenge, engaging experienced recruiters might alleviate some of these challenges, but it is unlikely to resolve them all. The diverse and often conflicting expectations between RFOs and the public, coupled with the difficulty of accommodating participants' varying needs and vulnerabilities, make managing commitments and expectations a significant hurdle. Ensuring meaningful dialogue and equal engagement is complicated by power imbalances and the need for effective mediation. Accommodating vulnerable groups requires a nuanced understanding and sensitive approach, which can be resource-intensive. Formulating funding calls that balance scientific and social relevance involves navigating complex stakeholder dynamics. The lack of expertise in engagement ethics further complicates the process, necessitating external input and ongoing learning. Lastly, the unpredictability of resource constraints and external factors such as regulatory requirements adds another layer of complexity. These multifaceted challenges necessitate adaptive, context-specific strategies and highlight the ongoing need for innovative solutions to foster ethical and effective public engagement in research funding.

To illustrate these challenges, we use the example of a German RFO (VDI/VDE Innovation + Technik GmbH, hereafter ‘VDI/VDE’) tasked with developing a call for project proposals to support informal caregivers through interactive technologies. The RFO set up a citizen advisory board consisting of 15 caregivers, responsible for evaluating and selecting proposals. This RFO faced several challenges, including difficulty in recruiting diverse caregivers, managing expectations and commitments, and accommodating vulnerabilities.

Recruiting caregivers was difficult due to selection bias (challenge 1) and potential participants' previous negative experiences with administrative bodies. Time constraints limited the input some caregivers could provide, exacerbating knowledge-based power imbalances. Additionally, mismatching expectations and rigid protocols demotivated some participants (challenge 2). What is more, joining the advisory board would inhibit them from providing care and would thus put their patient(s) at risk (challenge 4). Few caregivers could afford a substitute caregiver and as a response, VDI/VDE reimbursed any care expenses that caregivers incurred. Compensation was also available for travel and accommodation costs. Nevertheless, the RFO’s administrative processes caused substantial delays – of up to several weeks – in the reimbursements, again imposing a financial burden on some of the caregivers.

Also, some board members were consequently more familiar with the projects than others, thus exacerbating knowledge-based power imbalances (challenge 3). Several caregivers were therefore unable to convincingly voice their opinions, and a few acknowledged feeling undervalued or even intimidated. Other caregivers deemed their responsibilities too limited and pleaded for greater influence when mentoring projects. For example, some caregivers provided their professional expertise (i.e., knowledge of IT and engineering) even though they were invited by the board to provide their experiences as caregivers. As a result, rigid protocols (challenge 7) and mismatching expectations (challenge 2) may have demotivated some participants.

This real-life case illustrates how a single public engagement process by a research and innovation funding organization can encounter multiple, overlapping challenges simultaneously. It underscores the importance of providing targeted guidance to research and innovation funders on how to effectively navigate and address these diverse challenges in public engagement. The following section will provide actionable recommendations to help research and innovation funders effectively address these challenges and foster more inclusive, ethical, and efficient public engagement practices.

## Discussion

The seven challenges identified from the aggregated feedback received by the RFOs during their public engagement processes underscore the need for context-specific guidance. Each difficulty encountered necessitates careful attention and scrutiny tailored to the specific circumstances of each case.

European RFOs are increasingly experimenting with public engagement in funding processes, aspiring to uphold ethical values such as justice, equality, and safety. Despite the benefits, challenges related to ethical public engagement persist, requiring context-adaptive insights and tools to meaningfully and inclusively engage the public. Despite decades of research on ethics and upstream engagement, RFOs still face challenges that are too context-specific to be addressed in a standardized manner. These challenges create gaps between how public engagement in research funding *should* be organized and *can* be organized.

Based on the analysis of our seven challenges faced by RFOs in ethical (therefore also inclusive) public engagement, several recommendations can be made to improve their current practices: see Table 1, below.

**Table 1.** Recommendations for ethical public engagement in response to the challenges detected.

Challenges	Recommendations
Recruiting participants	Recruitment strategies with experienced recruiters and community organizations
Commitments and expectations	Clear communication of roles, expectations, and outcomes through codes of conduct
Meaningful dialogue and equal engagement	Training of mediators to address power imbalances
Accommodating vulnerability	Flexible engagement methods and tailored support
Funding call formulations	Collaborative feedback loops for inclusive funding call formulation
Lack of expertise in engagement ethics	Enhancing ethical standards through internal expertise and external advisory inputs
Uncertainty, resource constraints, and external factors	Developing adaptive strategies for flexible and ethical public engagement

### Recommendation 1: recruitment strategies with experienced recruiters and community organizations

RFOs should develop comprehensive recruitment strategies that leverage the expertise of professional recruiters, social scientists, and local community organizations to ensure diverse and representative participation. Social scientists, for instance, can play a dual role: not only in designing recruitment strategies that reduce bias but also in brokering partnerships between stakeholders and facilitating effective engagement processes. These strategies should include thorough analyses to identify underrepresented groups, implementing outreach programs tailored to the specific needs and preferences of diverse populations, offering incentives and removing participation barriers such as providing compensation for the time dedicated. Additionally, this would involve establishing long-term relationships with community leaders and organizations to build trust and ensure sustained engagement.

### Recommendation 2: Clear communication of roles, expectations, and outcomes through codes of conduct

RFOs should establish and disseminate detailed codes of conduct that clearly define the roles, expectations, and outcomes for all participants. This would typically include hosting preliminary orientation sessions to explain the engagement process and the targeted objectives. Additionally, it would involve providing written guidelines to ensure all participants understand their commitment and the impact of their involvement, and regularly updating participants on progress and outcomes. Furthermore, creating feedback mechanisms to allow all participants to voice any potential concerns or suggestions would ensure ongoing alignment and adaptation of expectations on both sides.

### Recommendation 3: training of mediators to address power imbalances

RFOs should invest in comprehensive training for mediators to foster equitable participation, or perhaps outsource this activity. This includes providing mediators with proper training in conflict resolution, active listening, and cultural competencies. To achieve balanced participation, structured dialogue techniques are essential. Examples of such techniques might include small group discussions, thematic warm-ups, or breakout sessions, depending on the context. A wide variety of other methods exist and the appropriate approach depends on the specific engagement scenario. Regular assessment and refinement of mediation strategies through participant feedback and mediator reflections would also be essential.

### Recommendation 4: flexible engagement methods and tailored support

RFOs should design flexible engagement methods that accommodate the needs of vulnerable groups by offering various participation formats (e.g., in-person, virtual, hybrid), so as to take into consideration different preferences and constraints. This would imply providing tailored support such as sign language interpreters, accessible venues, and assistive technologies. Additionally, ensuring financial compensation for participants' time and expenses could also be beneficial, as is establishing dedicated support teams to assist vulnerable participants throughout the engagement process.

### Recommendation 5: collaborative feedback loops for inclusive funding call formulation

RFOs should implement collaborative feedback loops that integrate input from public participants and traditional stakeholders at multiple stages of the funding call formulation process. This could be achieved by conducting joint workshops and focus groups to co-create funding priorities and call criteria. Additionally, implementing iterative review cycles where drafts of funding calls are shared with stakeholders for feedback and improvement would be beneficial. Utilizing digital platforms to facilitate continuous input and collaboration would help ensure all voices are heard. Furthermore, ensuring transparency in how public input is integrated into the final funding calls, with clear explanations of the final decisions made, would also contribute to this aim.

### Recommendation 6: enhancing ethical standards through internal expertise and external advisory inputs

RFOs can enhance their ethical standards by fostering professionalism and embracing core values such as fairness, equality, and respect in their public engagement activities. Rather than establishing dedicated engagement ethics committees, which might overlap with the role of research ethics committees, RFOs can adopt streamlined processes that ensure ethical public engagement while maintaining flexibility.

This can be achieved by providing ongoing ethics training for staff involved in public engagement activities and fostering a culture of ethical awareness. Collaborating with external advisory boards or ethicists can be useful to review and enhance engagement strategies where needed, helping ensure continuous alignment with ethical principles. Regular reviews of engagement practices, through flexible audits or assessments, can support ongoing improvements and maintain high standards of ethical engagement.

If more structured oversight is considered beneficial, RFOs could explore establishing a shared advisory board accessible to multiple organizations, ensuring a consistent approach while avoiding duplication of efforts across individual RFOs.

### Recommendation 7: developing adaptive strategies for flexible and ethical public engagement

RFOs should develop adaptive strategies to manage uncertainty and resource constraints while maintaining ethical standards. This target could be achieved by creating flexible engagement frameworks that can be adjusted based on emerging needs and feedback, to address unforeseen challenges and ensure the continuity of engagement activities, to monitor and respond to external factors (e.g., regulatory changes, societal shifts), and regularly reviewing and updating engagement plans to incorporate lessons learned and best practices.

Implementing these practical recommendations could help RFOs bridge the existing gaps, in view of ethical public engagement. This could in turn contribute to fostering more ethical and effective funding practices. These findings should be considered with an understanding of their limited generalizability, given our purposive sampling strategy.

## Concluding remarks

European Research Funding Organizations (RFOs) face multiple, interrelated challenges in executing ethical public engagement. These challenges stem from recruiting a representative public, managing diverse commitments and expectations, ensuring meaningful dialogue, accommodating vulnerable participants, formulating inclusive funding calls, and addressing a lack of engagement ethics expertise—all within the constraints of limited resources and external pressures. To bridge these gaps, RFOs must develop context-specific strategies and adopt adaptive, flexible approaches that prioritize ethical standards and inclusivity.

In response to these challenges, we have provided recommendations for each of the challenges. These seven recommended practices—leveraging experienced recruiters, clear communication through codes of conduct, mediator training, flexible engagement methods, collaborative feedback loops, enhancing ethical standards, and developing adaptive strategies—could help RFOs enhance their engagement practices. These improvements can lead to more ethical, and effective public participation, ultimately fostering a more just and equitable research funding process.

Further, RFOs can draw on established resources and experiences from organizations in fields such as health and social care, as well as from international examples, to guide and enhance their public involvement strategies. Collaborative efforts between academia, funders, and public involvement practitioners can play a critical role in addressing these persistent challenges. Iterative learning and cross-sectoral cooperation will help RFOs bridge the gap between the ideal and practical organization of public engagement, ensuring that ethical, and effective practices become embedded in the research funding process.

## Ethical approval and consent

Ethical approval and consent were not required.

## Data Availability

The primary data discussed in this paper, which served as the basis for our analysis and reflections, originates from the Pilots of PRO-Ethics, an H2020 project. These Pilots were internal to the consortium, and the results are publicly available on the project's website. See: https://pro-ethics.eu/outputs; and https://pro-ethics.eu/pilots/
