# Supplementary material for: Challenges to ethical public engagement in research funding: a perspective from practice
Source: Open Res Eur. 2024 Nov 6;4:179. Originally published 2024 Aug 13. [Version 2] doi: 10.12688/openreseurope.18126.2 (PMC11549538; doi:10.12688/openreseurope.18126.2)
Supplement: Peer review history

# Reviewer report: Reviewer response for version 2

**DOI:** 10.21956/openreseurope.20362.r45900

**Published:** 2024-11-08

**Authors:** Loreta Tauginienė

**Affiliations:** 1 Kazimieras Simonavicius University, Vilnius, Lithuania

Thank you for the opportunity to evaluate the revised manuscript.

 The suggestions were well addressed.

Is the case presented with sufficient detail to be useful for teaching or other practitioners?

Yes

Is the work clearly and accurately presented and does it cite the current literature?

No

If applicable, is the statistical analysis and its interpretation appropriate?

Not applicable

Are all the source data underlying the results available to ensure full reproducibility?

No

Are the conclusions drawn adequately supported by the results?

Yes

Is the background of the case’s history and progression described in sufficient detail?

No

Reviewer Expertise:

citizen science, social responsibility, RRI, research ethics, research integrity, public engagement

I confirm that I have read this submission and believe that I have an appropriate level of expertise to confirm that it is of an acceptable scientific standard.

# Reviewer report: Reviewer response for version 2

**DOI:** 10.21956/openreseurope.20362.r45899

**Published:** 2024-11-08

**Authors:** Lucy Carter

**Affiliations:** 1 CSIRO Environment, Brisbane, Queensland, Australia

Thank you to the authorship team who've provided comprehensive responses to reviewers' remarks. The paper is much more impactful now.

Is the case presented with sufficient detail to be useful for teaching or other practitioners?

Yes

Is the work clearly and accurately presented and does it cite the current literature?

Partly

If applicable, is the statistical analysis and its interpretation appropriate?

Not applicable

Are all the source data underlying the results available to ensure full reproducibility?

No source data required

Are the conclusions drawn adequately supported by the results?

Partly

Is the background of the case’s history and progression described in sufficient detail?

Partly

Reviewer Expertise:

Social science; engagement research and practice; interdisciplinary research approaches

I confirm that I have read this submission and believe that I have an appropriate level of expertise to confirm that it is of an acceptable scientific standard.

# Reviewer report: Reviewer response for version 1

**DOI:** 10.21956/openreseurope.19594.r44961

**Published:** 2024-10-21

**Authors:** Loreta Tauginienė

**Affiliations:** 1 Kazimieras Simonavicius University, Vilnius, Lithuania

The article is interesting and relevant to practitioners and other readers. It is well-structured and reads with ease.

 Here are some suggestions for further improvement.

**Section 1. **The readers might benefit from the concise literature review on public engagement (PE) in funding from a broader perspective (e.g. practice of municipalities). This might be accommodated either in the Section 1 or made as a separate section. Please also consider including the latest findings on PE in research funding.

**Section 2.** It is unclear how challenges were identified in methodological terms. What were methods of data collection used? How was the data collected analyzed? Any limitations? Then, research questions are unclear.

 The relevance and scope of each challenge is well described combining data of the project partners with supportive references.

**Section 3.** The real-life case is well-described. It addresses most of challenges. Any lessons learned that might be useful for the next section on recommendations?

**Section 4. **It is suggested considering adding "and inclusive" ( *ethical and inclusive engagement*) because it appears to have the same importance here.

**Other observations:** Statements and Declarations should be complemented with *Conflict of Interest*.

Is the case presented with sufficient detail to be useful for teaching or other practitioners?

Yes

Is the work clearly and accurately presented and does it cite the current literature?

No

If applicable, is the statistical analysis and its interpretation appropriate?

Not applicable

Are all the source data underlying the results available to ensure full reproducibility?

No

Are the conclusions drawn adequately supported by the results?

Yes

Is the background of the case’s history and progression described in sufficient detail?

No

Reviewer Expertise:

citizen science, social responsibility, RRI, research ethics, research integrity, public engagement

I confirm that I have read this submission and believe that I have an appropriate level of expertise to confirm that it is of an acceptable scientific standard, however I have significant reservations, as outlined above.

# Response

**Published:** 2024-10-25

**Authors:** Kalli GIANNELOS

**Affiliations:** Governance and Regulation Chair, Paris-Dauphine University, Paris, Île-de-France, France

Dear Reviewer,   We sincerely thank you for your detailed and constructive feedback. Your valuable insights have significantly contributed to improving our manuscript, and we are grateful for the opportunity to address them. Below is a summary of the revisions made in response to your suggestions:  

***Comment:*** *"Section 1. The readers might benefit from the concise literature review on public engagement (PE) in funding from a broader perspective (e.g. practice of municipalities). This might be accommodated either in the Section 1 or made as a separate section. Please also consider including the latest findings on PE in research funding."*

**Response:** Thank you for highlighting this. We have added a paragraph in Section 1 that provides a concise literature review on public engagement in funding, with a focus on broader examples such as participatory budgeting, which is a key area of study. Additionally, we clarified the current state of research on public engagement in research funding, emphasizing the prevalence of studies in the health and biomedical sectors. This important finding supports the relevance of our work, as it broadens the existing scholarly literature by examining public engagement in research and innovation funding across a wider range of sectors.

***Comment:*** *"Section 2. It is unclear how challenges were identified in methodological terms. What were methods of data collection used? How was the data collected analyzed? Any limitations? Then, research questions are unclear. The relevance and scope of each challenge is well described combining data of the project partners with supportive references."* **Response: **The revised text at the beginning of section 2 now clarifies the methodology used for both data collection and analysis, outlines the research question, and a sentence at the end of the Discussion (section 4) mentions the limitations of this approach.  

***Comment:*** *"Section 3. The real-life case is well-described. It addresses most of challenges. Any lessons learned that might be useful for the next section on recommendations?"* **Response: **In response, we have added a section discussing the lessons learned from the real-life case. This addition highlights how a single public engagement process by a research and innovation funding organization can face multiple, overlapping challenges simultaneously. We have also emphasized the need for providing targeted guidance to funders on how to effectively navigate these challenges to ensure more inclusive, ethical, and efficient public engagement practices (which is addressed in the subsequent section).  

***Comment:*** *"Section 4. It is suggested considering adding "and inclusive" (ethical and inclusive engagement) because it appears to have the same importance here."*

**Response:** Thank you for your suggestion. At the beginning of Section 4, we have clarified that ethical public engagement inherently includes inclusivity, emphasizing that a public engagement process cannot be considered ethical without being inclusive. This distinction highlights 'ethical' as an overarching category that encompasses inclusivity.    

***Comment:*** *"Other observations: Statements and Declarations should be complemented with Conflict of Interest."*

**Response: **There are no conflicts of interest related to this paper. The Statements and Declarations section also addresses the typical concerns regarding competing interests, ethical approval, and data availability.   We hope these revisions adequately address your concerns and further improve the clarity and relevance of the manuscript. Once again, thank you for your invaluable feedback.

# Reviewer report: Reviewer response for version 1

**DOI:** 10.21956/openreseurope.19594.r43871

**Published:** 2024-10-10

**Authors:** Sarah Hatch

**Affiliations:** 1 School of Medicine, Cardiff University, Cardiff, Wales, UK

Thank you for providing an opportunity to review this paper. I feel this case study offers important learning points for the research community. I hope the following comments are helpful to the authors in strengthening this work.

 Regarding terminology used in this case study – be mindful that in certain sectors and in different countries ‘public engagement’ can mean different things to different people. There is a lot of literature available on this. In health and social care research in the UK, for example, public involvement and public engagement are both used and mean different things. At the outset of the paper, it maybe helpful to define your meaning of ‘public engagement’ to improve understanding across disciplines, sectors, countries etc.

 The seven highlighted challenges exist for a much wider range of organizations and have a much broader scope than just Research Funding Organisations.

 Challenge 1 – recruiting members of the public to get involved in any aspect of research is a challenge for the research community as a whole not just for RFOs. There is growing literature on how to encourage a more diverse public to get involved in the research cycle process which would be relevant to RFOs or any organisation/researcher wanting to involve members of the public in their work.

 When working with health and care researchers, the priority is to attract representation from members of the public who have ‘lived experience’ of a particular health condition/social care aspect that is being researched. Certain essential criteria need to be clearly articulated in a role description which then can be promoted widely to identified key stakeholders including charities; community groups; libraries; companies who facilitate contact with hard-to-reach groups.

 For RFOs, fund scope/area will help to inform appropriate representation, so selection is not just based on different socio-economic factors.

 There are a range of resources out there (that could be referenced) to support inclusivity in research. For example, Reaching Out: A guide to being inclusive in public involvement (learningforinvolvement.org.uk)

 In the UK, we also have the UK Standards for Public Involvement which include six standards which make up a framework for what good public involvement in research looks like. These can be adapted to different situations and are designed to be used as a guide/tool to help researchers and research organisations identify what they are doing well and what needs improving. The six standards are focussed on Inclusive Opportunities; Working Together; Impact; Governance; Communications; Support and Learning. Similar guidance maybe available in other European countries?

 Availability of budget often dictates how much involvement will be possible (numbers of people to be recruited to take part etc).  

 Challenge 2 is included in the ‘Working Together’ standard of the UK Standards for Public Involvement.

 Challenge 6

 Engagement ethics. Use of the word ‘ethics’ here could be confusing. Professionalism and adequate resource (human and financial) are key to delivering quality public engagement. This does not rely on specific expertise but practitioners who conduct themselves and their work in a professional manner and have access to adequate resource to support their engagement work.

 Challenges in practice: The case of an RFO

 ‘The lack of expertise in engagement ethics further complicates the process, necessitating external input and ongoing learning’.

 My concern here is that you are creating an unnecessary barrier to conducting public involvement/engagement? Creating a field of expertise ‘engagement ethics’ lends itself to being a specialised area requiring expert knowledge. I believe that this is potentially misleading. All working professionals should take responsibility for going about their work in a professional and ethical manner embracing values of ‘respect’ ‘fairness’; and ‘transparency’. I worry that stating a ‘lack of engagement ethics expertise’ could easily be presented as an excuse for not doing public engagement work, effectively acting as an additional barrier. 

 Case Study – highlights ‘involvement’ challenges experienced across the sector. These are not new and happen outside of RFOs.

 ‘Ensuring financial compensation for participants' time and expenses could also be beneficial’ - To support inclusivity offering compensation (financial or non-financial) is a recognised UK Standard for public involvement that all researchers/organisations should adopt when planning a public involvement activity with patients and members of the public.

 Recommendation 6 - **Enhancing ethical standards through internal expertise and external advisory inputs**

 Once again, my concern here is that this could potentially create an unnecessary barrier and should not be needed if organisations operate professionally embracing values of fairness, equality, respect etc.

 Ethical public engagement – This term raises several queries:-

- I fear the use of this term will cause confusion in a growing debate concerning Public Involvement and Engagement versus Research Ethics (from higher education institutional research ethics committees). Public involvement and public engagement in research does not require research ethics but should be planned and conducted in a professional manner embracing EDI principles and taking into account risk. Public Involvement and Engagement professionals working alongside academic colleagues ensure that public involvement and engagement activity is delivered in a way that meets the recognised quality (UK) standards and plans and prepares for any risks associated with the activity. There is a growing debate around creating a recognised simple streamlined process acceptable to journal editors and funders to avoid researchers having to put their public engagement and involvement activity unnecessarily through local University Research Ethics Committees.
- Recommending the creation of dedicated engagement ethics committees could be confused with research ethics committees and appear overly burdensome.
- Would they be necessary in all RFOs?  Would the level of engagement in RFOs warrant the establishment of dedicated ‘engagement’ ethics committee? Could a simpler process be put in place? If deemed necessary, potentially an overarching Committee accessible to all RFOs would be a more effective solution, avoiding duplication of effort across RFOs.

Resources developed by academia, funders and the public in the UK to address the identified challenges (UK Standards for Public Involvement) to public involvement in research would be equally applicable and helpful to RFOs working to engage the public in the formulation of funding calls. Resources from other countries may also exist and be helpful here.

 Several funding bodies in the health and social care field have embedded public involvement in their funding processes (National Institute of Health Research; Health Research Authority; Health and Care Research Wales) as well as health charities (Cancer Research UK; Diabetes UK). European RFOs could potentially learn from the experience of these organisations in guiding and embedding patient and public involvement into organisational processes.

 I hope my review comments are helpful. Happy to respond to any author queries.

Is the case presented with sufficient detail to be useful for teaching or other practitioners?

Yes

Is the work clearly and accurately presented and does it cite the current literature?

Partly

If applicable, is the statistical analysis and its interpretation appropriate?

Not applicable

Are all the source data underlying the results available to ensure full reproducibility?

Yes

Are the conclusions drawn adequately supported by the results?

Partly

Is the background of the case’s history and progression described in sufficient detail?

Partly

Reviewer Expertise:

Patient and Public Involvement and Engagement Professional in field of health research and education.

I confirm that I have read this submission and believe that I have an appropriate level of expertise to confirm that it is of an acceptable scientific standard, however I have significant reservations, as outlined above.

# Response

**Published:** 2024-10-25

**Authors:** Kalli GIANNELOS

**Affiliations:** Governance and Regulation Chair, Paris-Dauphine University, Paris, Île-de-France, France

Dear Reviewer,   We sincerely appreciate your thorough and constructive feedback. Your insights have been invaluable in refining our manuscript, and we are grateful for the opportunity to incorporate them. Below is a summary of the revisions made in response to your comments: ***Comment:*** *"Regarding terminology used in this case study – be mindful that in certain sectors and in different countries ‘public engagement’ can mean different things to different people. There is a lot of literature available on this. In health and social care research in the UK, for example, public involvement and public engagement are both used and mean different things. At the outset of the paper, it maybe helpful to define your meaning of ‘public engagement’ to improve understanding across disciplines, sectors, countries etc."*

**Response**: The introduction has been revised to clarify the meaning of “public engagement” within the context of this study. We acknowledge that the term may vary across sectors and countries, and this definition aims to ensure clarity for readers from different disciplines. ***Comment:*** *"The seven highlighted challenges exist for a much wider range of organizations and have a much broader scope than just Research Funding Organisations."*

**Response: **We have expanded the manuscript to reflect that the seven challenges identified are experienced by a wide range of organizations beyond just RFOs. This change has been made in section 2 to ensure the broader relevance of our findings.  

***Comment:*** *"Challenge 1 – recruiting members of the public to get involved in any aspect of research is a challenge for the research community as a whole not just for RFOs. There is growing literature on how to encourage a more diverse public to get involved in the research cycle process which would be relevant to RFOs or any organisation/researcher wanting to involve members of the public in their work.*

*When working with health and care researchers, the priority is to attract representation from members of the public who have ‘lived experience’ of a particular health condition/social care aspect that is being researched. Certain essential criteria need to be clearly articulated in a role description which then can be promoted widely to identified key stakeholders including charities; community groups; libraries; companies who facilitate contact with hard-to-reach groups. For RFOs, fund scope/area will help to inform appropriate representation, so selection is not just based on different socio-economic factors. There are a range of resources out there (that could be referenced) to support inclusivity in research. For example,* *Reaching Out: A guide to being inclusive in public involvement (learningforinvolvement.org.uk)* *. In the UK, we also have the UK Standards for Public Involvement which include six standards which make up a framework for what good public involvement in research looks like. These can be adapted to different situations and are designed to be used as a guide/tool to help researchers and research organisations identify what they are doing well and what needs improving. The six standards are focussed on Inclusive Opportunities; Working Together; Impact; Governance; Communications; Support and Learning. Similar guidance maybe available in other European countries? Availability of budget often dictates how much involvement will be possible (numbers of people to be recruited to take part etc)."*

**Response: **In response to your feedback, we have broadened the discussion of recruitment in Challenge 1 to acknowledge that this issue extends beyond RFOs. We have also incorporated references to relevant literature on recruitment diversity and inclusivity, including frameworks from health and social care research.    

***Comment:*** *"Challenge 6. Engagement ethics. Use of the word ‘ethics’ here could be confusing. Professionalism and adequate resource (human and financial) are key to delivering quality public engagement. This does not rely on specific expertise but practitioners who conduct themselves and their work in a professional manner and have access to adequate resource to support their engagement work. Challenges in practice: The case of an RFO. ‘The lack of expertise in engagement ethics further complicates the process, necessitating external input and ongoing learning’. My concern here is that you are creating an unnecessary barrier to conducting public involvement/engagement? Creating a field of expertise ‘engagement ethics’ lends itself to being a specialised area requiring expert knowledge. I believe that this is potentially misleading. All working professionals should take responsibility for going about their work in a professional and ethical manner embracing values of ‘respect’ ‘fairness’; and ‘transparency’. I worry that stating a ‘lack of engagement ethics expertise’ could easily be presented as an excuse for not doing public engagement work, effectively acting as an additional barrier."*

**Response**: We have revised Challenge 6 to clarify that ethical public engagement does not necessitate the creation of a specialized field. Instead, the revision highlights the role of professionalism, fairness, and transparency in guiding ethical engagement, avoiding unnecessary barriers.    

***Comment:*** *"Ensuring financial compensation for participants' time and expenses could also be beneficial’ - To support inclusivity offering compensation (financial or non-financial) is a recognised UK Standard for public involvement that all researchers/organisations should adopt when planning a public involvement activity with patients and members of the public."* **Response**: Your suggestion regarding compensation for participants has been addressed. The text in Challenge 4 has been revised to emphasize that compensation is a recognized standard for inclusive public engagement, with examples drawn from established UK practices and beyond.    

***Comment:*** *"Recommendation 6 - Enhancing ethical standards through internal expertise and external advisory inputs. Once again, my concern here is that this could potentially create an unnecessary barrier and should not be needed if organisations operate professionally embracing values of fairness, equality, respect etc. Ethical public engagement – This term raises several queries:*

- *I fear the use of this term will cause confusion in a growing debate concerning Public Involvement and Engagement versus Research Ethics (from higher education institutional research ethics committees). Public involvement and public engagement in research does not require research ethics but should be planned and conducted in a professional manner embracing EDI principles and taking into account risk. Public Involvement and Engagement professionals working alongside academic colleagues ensure that public involvement and engagement activity is delivered in a way that meets the recognised quality (UK) standards and plans and prepares for any risks associated with the activity. There is a growing debate around creating a recognised simple streamlined process acceptable to journal editors and funders to avoid researchers having to put their public engagement and involvement activity unnecessarily through local University Research Ethics Committees.*
- *Recommending the creation of dedicated engagement ethics committees could be confused with research ethics committees and appear overly burdensome.*

*Would they be necessary in all RFOs?  Would the level of engagement in RFOs warrant the establishment of dedicated ‘engagement’ ethics committee? Could a simpler process be put in place? If deemed necessary, potentially an overarching Committee accessible to all RFOs would be a more effective solution, avoiding duplication of effort across RFOs."*

**Response**: In line with your suggestion, we have revised Recommendation 6 to avoid the creation of dedicated engagement ethics committees. Instead, we propose more flexible ethical processes, with the possibility of a shared advisory board to provide structured oversight if needed.    

***Comment:*** *"* *Resources developed by academia, funders and the public in the UK to address the identified challenges (UK Standards for Public Involvement) to public involvement in research would be equally applicable and helpful to RFOs working to engage the public in the formulation of funding calls. Resources from other countries may also exist and be helpful here.*

*Several funding bodies in the health and social care field have embedded public involvement in their funding processes (National Institute of Health Research; Health Research Authority; Health and Care Research Wales) as well as health charities (Cancer Research UK; Diabetes UK). European RFOs could potentially learn from the experience of these organisations in guiding and embedding patient and public involvement into organisational processes."* **Response**: We have integrated references to international resources and experiences that can inform and enhance public engagement strategies. The revised conclusion emphasizes the importance of cross-sectoral and international collaboration to address these challenges.   We trust these revisions address your concerns and enhance the clarity and relevance of the manuscript. Thank you once again for your invaluable feedback.

# Reviewer report: Reviewer response for version 1

**DOI:** 10.21956/openreseurope.19594.r43879

**Published:** 2024-09-12

**Authors:** Lucy Carter

**Affiliations:** 1 CSIRO Environment, Brisbane, Queensland, Australia

The paper offers insights into the challenges faced by European RFOs in conducting ethical public engagement activities. While the main point being made concerns the lack of governance around how RTOs plan and deliver good public engagement, the challenges are not only specific to RFOs but to all research partnerships. The recommendations the authors make are laudable, and draw from existing literature. I did wonder whether the recommendations would be mute in other non-European contexts given the different institutional models globally. 

 I offer some suggestions for refinement in the paper below:

**A little more explanation is needed please on: **The methods used to collate the identified 7 challenges. It was unclear whether these were generated from a series of discussions/reflections. A little more detail is needed in terms of the data and the analysis that went into formulating these challenges. Perhaps 2-3 lines would be enough.

**Introduction, para 1, final sentence:** Just be careful here, communication and consultation processes almost always grant no power to publics (i.e. these methods are often one-way, top-down processes where empowerment is unlikely) See this useful spectrum from IAP2 https://www.iap2.org/page/SpectrumEvolution

**Final paragraph under Challenge 1**: I’m not convinced of the selection bias argument. Sampling-related bias can be reduced but never eliminated. It’s also likely to be encountered by staff working in RFOs as well as experienced recruiters. Suggest that the authors make a stronger argument here by simply advocating for inclusion of appropriate expertise in social research (like social scientists, or similar) to design research processes that RFOs undertake. You could also argue that independent research expertise would likely reduce bias of other kinds, as well as perceived conflicts of interest.

**Challenge 3** would benefit from inclusion of some references.

**Recommendation 1**: I would add ‘social scientists’ to the mix of recommended experts. This group can also broker partnerships and facilitate engagement processes.

**Recommendation 3:** Here you list several specific methods. While these might be OK, they might also not be the right ones for the context. Perhaps just add a statement here that these are examples. In any qualitative methods textbook, you would find dozens of method examples, all designed for diverse scenarios.

**Finally,** As an Australian researcher, our RFOs contract out almost all deliberate public engagement initiatives to science or university organisations, sometimes in partnership. While some Australian RFOs may conduct consultation-type engagement on their own, historically, deeper engagement has been conducted by research organisations and designed and delivered by expert researchers. Under this model, research ethics approval processes along with academic and engagement expertise ensures the design of quality research processes.

 The concept of RFOs conducting deep research themselves is foreign in the research ecosystem in Australia, and much of the Indo-Pacific region. It was difficult for me to understand this difference in research governance in the paper initially and so I wondered whether it was possible to clarify somewhere in the introduction that globally, there are other research governance models in play (and hence where your identified challenges might not be relevant.)   

 Happy to respond to any author queries about this review.

Is the case presented with sufficient detail to be useful for teaching or other practitioners?

Yes

Is the work clearly and accurately presented and does it cite the current literature?

Partly

If applicable, is the statistical analysis and its interpretation appropriate?

Not applicable

Are all the source data underlying the results available to ensure full reproducibility?

No source data required

Are the conclusions drawn adequately supported by the results?

Partly

Is the background of the case’s history and progression described in sufficient detail?

Partly

Reviewer Expertise:

Social science; engagement research and practice; interdisciplinary research approaches

I confirm that I have read this submission and believe that I have an appropriate level of expertise to confirm that it is of an acceptable scientific standard, however I have significant reservations, as outlined above.

# Response

**Published:** 2024-10-25

**Authors:** Kalli GIANNELOS

**Affiliations:** Governance and Regulation Chair, Paris-Dauphine University, Paris, Île-de-France, France

Dear Reviewer, We would like to sincerely thank you for your insightful and constructive feedback. Your comments have been invaluable in helping us refine the manuscript, particularly by clarifying key points and strengthening the arguments presented. Below is a summary of the revisions made in response to your critique:  

**Comment**: "A little more explanation is needed please on: The methods used to collate the identified 7 challenges. It was unclear whether these were generated from a series of discussions/reflections. A little more detail is needed in terms of the data and the analysis that went into formulating these challenges. Perhaps 2-3 lines would be enough."

**Response**: You raised concerns about the clarity regarding how the seven challenges were generated. To address this, we have revised the second section to provide additional details, explaining that the challenges were identified through a series of cross-learning workshops and discussions among the eight participating RFOs. We also clarified that the data was analyzed using an inductive thematic analysis. Verbatim transcriptions were coded at the sentence and paragraph level, providing the foundation for identifying the key themes and challenges.  

**Comment**: "Introduction, para 1, final sentence: Just be careful here, communication and consultation processes almost always grant no power to publics (i.e. these methods are often one-way, top-down processes where empowerment is unlikely) See this useful spectrum from IAP2 https://www.iap2.org/page/SpectrumEvolution"

**Response**: To highlight the fact that communication and consultation processes often do not grant significant power to the public, the manuscript now includes a reference to the IAP2 Public Participation Spectrum, clarifying that communication and consultation are often one-way, top-down mechanisms with limited public empowerment.  

**Comment**: "Final paragraph under Challenge 1: I’m not convinced of the selection bias argument. Sampling-related bias can be reduced but never eliminated. It’s also likely to be encountered by staff working in RFOs as well as experienced recruiters. Suggest that the authors make a stronger argument here by simply advocating for inclusion of appropriate expertise in social research (like social scientists, or similar) to design research processes that RFOs undertake. You could also argue that independent research expertise would likely reduce bias of other kinds, as well as perceived conflicts of interest."

**Response**: The original manuscript’s argument on selection bias was questioned, and it was suggested that social scientists or independent research experts could help in designing more robust research processes. The revised version of Challenge 1 now advocates for the inclusion of social scientists or independent research experts in the design of engagement processes to mitigate various forms of bias and potential conflicts of interest.  

**Comment**: "Challenge 3 would benefit from inclusion of some references."

**Response**: As recommended, we have updated Challenge 3 to incorporate relevant references that support the discussion on power imbalances and strategies for fostering meaningful dialogue. These citations substantiate the proposed methods for addressing knowledge-based power imbalances.  

**Comment**: "Recommendation 1: I would add ‘social scientists’ to the mix of recommended experts. This group can also broker partnerships and facilitate engagement processes." **Response**: The suggestion to include social scientists explicitly in Recommendation 1 has been implemented. Social scientists are now highlighted as key experts who not only contribute to recruitment strategies but also broker partnerships and facilitate effective engagement processes.  

**Comment**: "Recommendation 3: Here you list several specific methods. While these might be OK, they might also not be the right ones for the context. Perhaps just add a statement here that these are examples. In any qualitative methods textbook, you would find dozens of method examples, all designed for diverse scenarios."

**Response**: The original version of Recommendation 3 listed specific methods, which might not be universally appropriate. We have revised the text to clarify that the methods mentioned are examples and that a variety of other approaches may be more suitable depending on the engagement context.  

***Comment*** *: "Finally, As an Australian researcher, our RFOs contract out almost all deliberate public engagement initiatives to science or university organisations, sometimes in partnership. While some Australian RFOs may conduct consultation-type engagement on their own, historically, deeper engagement has been conducted by research organisations and designed and delivered by expert researchers. Under this model, research ethics approval processes along with academic and engagement expertise ensures the design of quality research processes.*

*The concept of RFOs conducting deep research themselves is foreign in the research ecosystem in Australia, and much of the Indo-Pacific region. It was difficult for me to understand this difference in research governance in the paper initially and so I wondered whether it was possible to clarify somewhere in the introduction that globally, there are other research governance models in play (and hence where your identified challenges might not be relevant.)."*

**Response**: It was noted that the challenges identified may not apply in non-European contexts due to different research governance models. To address this, we added a statement in the Introduction acknowledging that in regions such as Australia and the Indo-Pacific, RFOs often delegate public engagement to research organizations, making some of the challenges discussed less applicable in these areas.   The manuscript has been thoroughly revised in response to all the points raised in your review. We extend our gratitude once again for your thoughtful and detailed feedback, which has significantly enhanced the quality, clarity, relevance, and rigor of this work.
